# Case report of pulmonary epithelial trophoblastic tumor in a young female patient

**DOI:** 10.1097/MD.0000000000007097

**Published:** 2017-06-16

**Authors:** Lijun Jiang, Hongfei Xu, Yuan Lu, Weidong Li, Chengfei Zheng

**Affiliations:** a Department of Cardiothoracic Surgery; b Department of Vascular Surgery, First Affiliated Hospital of Zhejiang University, Hangzhou, China.

**Keywords:** beta-human chorionic gonadotropin, epithelial trophoblastic tumor, surgery

## Abstract

**Introduction:**

An epithelial trophoblastic tumor (ETT) is a kind of rare trophoblastic tumor that may have the correlation with a prior gestational event. Especially, the one that appears in the lung is extremely rare.

**Case summary:**

Here, we present a 24-year-old female patient with the chief complain of vaginal bleeding for more than 1 month, who was found to have a large mass (7.5 × 4.5 cm) in the right lower lobe, and it was finally confirmed as lung ETT by postoperative pathology after a successful radical resection of the pulmonary lobe.

**Conclusions:**

As the reason of an extreme rare occurrence of the ETT, doctors can easily misdiagnose the disease. When the patient was found to have a mass with irregular vaginal bleeding and a high level of beta-human chorionic gonadotropin, we need to consider ETT. Currently, surgery is still the most effective method.

## 1 Introduction

Epithelial trophoblastic tumor (ETT) is a rare trophoblastic tumor originating from chorionic-type intermediate trophoblasts.^[1]^ Shin and Kurman first described ETT in 1998. Less than 100 surgically treated cases have been reported in the English literature, and the ETT which appears in the lungs as an original lesion is even more rarely. ETT is a kind of trophoblastic tumor, which is different from a gestational trophoblastic tumor or a placental site trophoblastic tumor (PSTT).

## 2 Case report

**Figure 1:**
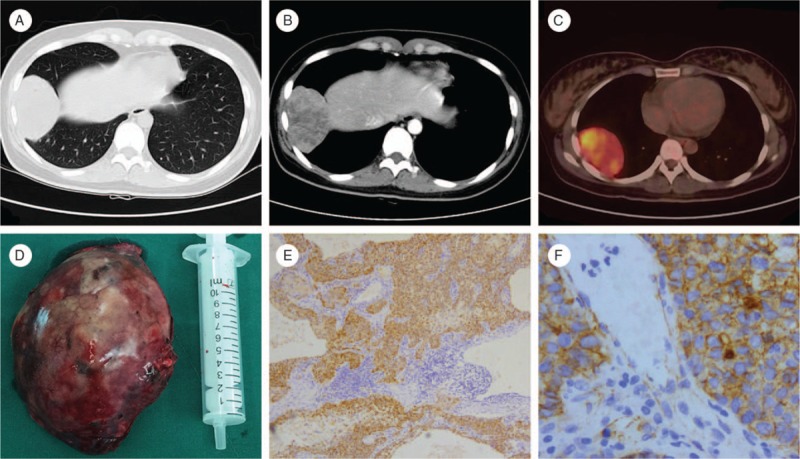
(A) CT revealed a lesion in the right lower lobe and the size was about 8.2 × 4.3 × 5.8 cm. (B) Contrast-enhanced CT revealed that the tumor with partial necrosis was not contrast-enhanced. (C) PET-CT showed a higher hypermetabolic lesion in the right lower lobe. (D) Gross pathologic examination revealed that the tumor (7.5 × 4.5 cm) was under the visceral pleura with high distension. (E) ETT cells arranged in the nest bulk (microscope ×100). (F) The ETT cells were markedly atypical and karyokinesis was increased (microscope ×400). CT = computed tomography, ETT = epithelial trophoblastic tumor, PET-CT = positron emission tomography-computed tomography.

A 24-year-old woman who had 4 histories of abortion had vaginal bleeding for more than 1 month. She was not eager to have a baby during the previous 4 abortions, so she did not get any drug treatment during and after those abortions. The level of serum beta-human chorionic gonadotropin (β-HCG) was 9075.4 mIU/mL. Ultrasound revealed an inhomogeneous mass (1.6 × 1.2 cm) close to the inner side of the right ovary, and a fluid sonolucent area (2.6 × 2.6 cm) can be seen in the pelvic cavity. Ectopic pregnancy was considered and the woman underwent laparoscopic salpingectomy on the right side with uterine curettage in a local hospital. After the operation, serum β-HCG level in patients had no significant decline. Then the patient came to our hospital, her physical examination was normal except for vaginal bleeding and fresh scars in abdomen, and computed tomography (CT) scanning revealed a lesion with partial necrosis in the right lower lobe of about 8.2 × 4.3 × 5.8 cm in size (Fig. 1A and B). Further examination of positron emission tomography-computed tomography (PET-CT) suggested a hypermetabolic tumor in the right lower lobe (Fig. 1C). In consideration of a malignant tumor, a right chest mass resection was done. Intraoperative frozen pathology revealed poorly differentiated carcinoma with negative lung cut edge, so a radical resection of pulmonary carcinoma was then operated. Postoperative pathology revealed that the tumor (7.5 × 4.5 cm) was under the visceral pleura (Fig. 1D). Microscopy showed that tumor cells were arranged in a nest bulk with obvious cell atypia and mitotic figures (Fig. 1E and F). The cutting edge of lung tissue was negative. Then we asked the patient's husband to make a pathological consultation for her previous diagnosis when our pathologists were in trapped with the diagnosis of this tumor, and the result showed that the previous diagnosis of normal fallopian tubes was correct. The final pathological diagnosis was of an epithelioid trophoblastic tumor. Immunoreactivity suggested a positive mark for P63, β-HCG, β-catenin, and Ki-67. The level of the postoperative serum β-HCG declined rapidly. The β-HCG decreased rapidly to normal and the vaginal bleeding disappeared after pulmonary surgery. The patient did not receive postoperative chemotherapy and 1-year follow-up of chest CT showed no evidence of tumor recurrence, as well as the level of the serum β-HCG was normal with no complications.

## 3 Discussion

ETT is a very rare tumor, which was first described by Shih and Kurman in 1998. As far as our knowledge, less than 100 surgically treated cases have been reported in the English literature. The onset age of ETT ranges from 15 to 48 years. The uterus is the most common primary site of ETT (40%), followed by the cervix (31%).^[2]^ ETT occurring outside of the reproductive system is rare, especially in lungs. The most common clinical manifestation of ETT is vaginal bleeding. Few patients manifest of abdominal distension, abdominal pain, or ascites. Common symptoms of ETT metastasis to lungs are cough, hemoptysis, etc. The level of serum β-HCG is usually moderately elevated (<2500 mIU/mL). Here we present a young female patient with 4 abortion history with the chief complain of vaginal bleeding for 1 month, who was misdiagnosed with an ectopic pregnancy at a local hospital and was finally proved to have primary lung ETT. Pathophysiologically, the cells of ETT are from chorionic-type intermediate trophoblast cell. Histologically, ETT is composed of nests of uniform mononucleated chorionic-type intermediate trophoblastic cells with eosinophilic or clear cytoplasm. As was seen in our case, positive p63 and β-HCG expression can help to distinguish ETT from PSTT and cervical squamous cell carcinoma.

Because of the low incidence of ETT, clinical understanding of ETT is insufficient. Such disease can be easily misdiagnosed as choriocarcinoma, ectopic pregnancy, or primary lung cancer. Choriocarcinoma is a type of cancer germ cell containing trophoblast cells that can make β-HCG. Its feature is the identification of intimately related syncytiotrophoblasts and cytotrophoblasts without any formation of definite placental type villi. Lung cancer like anaplastic carcinoma and small cell carcinoma can independently produce luteinizing hormone and β-HCG, which may cause painful bilateral or unilateral mammary gland hyperplasia. Generally, the main symptom of lung cancer is cough, hemoptysis, fever, and chest pain. X-ray or CT examinations are used to detect lung cancer and make definite diagnosis by histopathological examination. Ectopic pregnancy is a complication of pregnancy in which the embryo attaches outside the uterus. Dynamic monitoring of transvaginal ultrasonography and β-HCG is important for the detection of ectopic pregnancy.

Our patient was diagnosed with ectopic pregnancy in the local hospital, and she underwent a laparoscopy there. After surgery, vaginal bleeding and high level of β-HCG still existed. For this kind of patient, especially having the following characteristics: woman of reproductive age, history of pregnancy or abortion, elevated levels of β-HCG, irregular vaginal bleeding, and pulmonary mass, the diagnosis of pulmonary ETT should be considered.

Surgery and chemotherapy are the main treatment for a tumor. Though most of chemotherapy regimens, for example, EMA-CO, EMA-EP, or EMA, have been successfully used in the treatment of gestational trophoblastic neoplasia,^[3]^ the retrospective study from the literature shows that ETT seems to be resistant to combination chemotherapy regimens. Surgery is still the preferred treatment for the ETT at present. In this case, after complete resection of the tumor, β-HCG decreased rapidly to normal after pulmonary surgery, and the vaginal bleeding disappeared. So the patient did not receive postoperative chemotherapy. The patient recovered uneventfully after surgery and 1-year follow-up showed no evidence of recurrence.

## 4 Conclusions

An extrauterine epithelioid trophoblastic tumor of lung is extremely rare. Such disease can be easily misdiagnosed as choriocarcinoma, ectopic pregnancy, or primary lung cancer. For this kind of patient, especially having the following characteristics: woman of reproductive age, history of pregnancy or abortion, elevated levels of β-HCG, irregular vaginal bleeding, and pulmonary mass, the diagnosis of pulmonary ETT should be considered.
